# Chemoradiotherapy for Head and Neck Cancer

**DOI:** 10.3390/cancers15102820

**Published:** 2023-05-18

**Authors:** Ida D’Onofrio, Valerio Nardone, Alfonso Reginelli, Salvatore Cappabianca

**Affiliations:** 1 Department of Precision Medicine, University of Campania “L. Vanvitelli”, 80138 Naples, Italy; idado79@yahoo.it (I.D.); alfonso.reginelli@unicampania.it (A.R.); 2 Radiotherapy Unit, Ospedale del Mare, ASL Napoli 1 Centro, 80138 Naples, Italy

Head and neck squamous cell carcinoma (HNSCC) is a highly challenging cancer. Despite the advancements in treatment, the overall prognosis for HNSCC remains poor, with a five-year survival rate of around 50%. Due to its complexity and heterogeneity, the therapeutic response of HNSCC varies widely, regardless of clinicopathological risk factors. Various factors contribute to the heterogeneity of HNSCC, including genetic mutations, tumor microenvironment, and immune response. 

This Special Issue, which comprises seven papers (three original articles, two systematic reviews, and two reviews), presents knowledge on current evidence and future perspectives in the field of head and neck cancer chemoradiation treatment in both the clinical and translational research domains.

A prospective study conducted by D’Urso et al. [1] explored the role of magnetic resonance imaging (MRI) features as noninvasive biomarkers of disease progression in locally advanced oropharyngeal cancer, treated with definitive RT-CT. They found a statistically significant correlation between N diameter and disease control. Furthermore, DCE-MRI parameters, such as the Kep level both in primary tumors and lymph nodes and Ve in primary tumors, showed correlations with the disease outcome. The identification of predictive factors of therapeutic efficacy is a crucial research perspective to guide the personalization of radiotherapy treatment. 

PD1 is a molecule expressed on the surface of T lymphocytes, and it plays an important role in the modulation of T lymphocyte activity under normal physiological conditions. The blockade of PD1 and its ligand, PD-L1, represents an effective strategy to avoid the tumor escape process. Knowledge of the importance of the immune system in tumorigenesis and the introduction of new target therapies in recent decades, along with the increasing evidence of the role of immunity in the tumor response to radiotherapy, have allowed new strategies in cancer treatment to be detected. In both a murine model and a human clinical trial, Callejas-Valera et al. [2] demonstrated that PD1-PDL1 interaction may have a role in immune escape during chemoradiotherapy treatment for HNSCC. This study highlights that PD-1 blockade with concurrent cisplatin-based chemoradiotherapy improved the survival rate in murine models. In addition, they found that circulating PD-1+ T-cell counts dropped in a human clinical trial testing this therapeutic combination. Additional data suggested increases in the expression levels of other checkpoint markers, suggesting that further research is needed. 

Islam et al. [3] submitted a systematic review to this Special Issue that provided new insights into the identification of novel prognostic molecular biomarkers in nasopharyngeal cancer, which are involved in various biological pathways, including cancer proliferation, DNA damage repair, angiogenesis, hypoxia, epithelial-to-mesenchymal transition (EMT), differentiation, and immune response. The researchers identified seventeen biomarkers, including germline single-nucleotide polymorphisms (SNPs), methylation, microRNAs, mutation, and gene signatures in cancer and immune cells. These biomarkers could be investigated, in addition to known prognosticators in multi-center clinical trials, using AI technologies, with the aim to define a personalized treatment strategy, based on individualized clinical and biological characteristics.

Brachytherapy is an important option in head and neck squamous cell carcinoma (SCC) treatment strategies. Its use can be considered either exclusively or in combination with external radiotherapy, surgery, and/or chemotherapy. The essential requirements for brachytherapy are skills in the technique and the expertise of the radiation oncologist, as well as a close partnership between the surgeons and physicist. Tucek et al. [4] reviewed the role of brachytherapy in the treatment of early-stage oral cavity cancer in the modern era. The review provided an overview of the principles and historical background of brachytherapy. The main indications for brachytherapy are in the postoperative setting, exclusively or in combination with external radiotherapy in more advanced disease; in lip cancer, especially in elderly patients, leading to excellent oncologic and cosmetic results; and in local relapse/second tumors, above all in previously irradiated areas. This paper focused on recent improvements that have been made to the brachytherapy technique and the impact this technique has on the quality of life of patients.

Mounting evidence highlights the importance of germline variations in angiogenesis-regulating genes in cancer development and progression. Germline variations in genes that regulate angiogenesis can affect the balance between pro-angiogenic and anti-angiogenic factors, leading to abnormal blood vessel growth and potentially promoting cancer development and progression. This has been observed in various types of cancer, including ovarian, breast, and colorectal cancer, among others. Understanding the role of these genes in cancer can lead new diagnostic and therapeutic approaches. Butkiewicz et al. [5] tested their role as predictors of failure in the chemoradiation treatment of HNSCC. They found a correlation between some growth factors and extracellular matrix (ECM) components involved in angiogenesis and poor prognosis. These data could be used as an additive risk in the factor risk stratification and personalization of treatment in HNSCC.

The systematic review conducted by Di Rito et al. [6] analyzed 13 studies that assessed the effectiveness of postoperative radiochemotherapy (POCRT) in patients with minor risk factors in oral cavity carcinoma (OCC). The studies included patients with perineural invasion or lymphovascular invasion, pN1, DOI ≥ 5 mm close margin; node-positive level IV or V; pT3 or pT4; and multiple lymph nodes without ENE. The review suggested that the risk of disease recurrence in resected head and neck tumors with solely minor RF does not depend on a single risk factor (RF) but on the coexistence of multiple minor RFs. In fact, the simultaneous presence of multiple pathologic characteristics indicates a poor prognosis, even if these characteristics are not significant when analyzed separately; thus, this risk may be cumulative. However, further research is needed to determine the optimal timing, dose, and schedule of POCRT.

The management of HNSCC in elderly patients requires a comprehensive approach that considers both the features of the cancer and the patient’s overall health conditions. Clinical trials have under-represented elderly patients aged 65 and above, leading to a lack of clinical evidence for this cohort. Clinical management strategies for elderly HNSCC patients include the careful assessment of comorbidities and functional statuses, as well as the consideration of the risks and benefits of treatment. The review by Fasano et al. [7] emphasized the importance of using geriatric evaluation tools to identify potential barriers to treatment. Treatment options for HNSCC in elderly patients include radiation therapy and systemic therapies, both of which should be tailored to the individual patient’s needs and preferences. In this work, several schedules of radiotherapy were illustrated, including conventional fractionation, hypofractionation, and stereotactic body radiation therapy (SBRT), both in the radical and palliative setting. Moreover, the quality of life (QoL) in aged patients undergoing oncological treatment was assessed.

In summary, this Special Issue covers clinical and translational research related to HNSCC chemoradiation treatment, providing a complete understanding of the most recent advancements. We hope that healthcare professionals and researchers working in this field will find it useful.
